# Does upregulated host cell receptor expression provide a link between bacterial adhesion and chronic respiratory disease?

**DOI:** 10.1186/s12967-016-1063-x

**Published:** 2016-10-26

**Authors:** Ronan F. O’Toole, Shakti D. Shukla, Eugene H. Walters

**Affiliations:** School of Medicine, Faculty of Health, University of Tasmania, Medical Science 1, 17 Liverpool Street, Hobart, TAS 7000 Australia

**Keywords:** Airway epithelium, Platelet-activating factor receptor, Non-typeable *Haemophilus influenzae*, *Streptococcus pneumoniae*

## Abstract

Expression of the platelet-activating factor receptor is upregulated in the respiratory epithelium of smokers and chronic obstructive pulmonary disease patients. We have recently determined that increased expression of PAFr correlates with higher levels of adhesion to human bronchial epithelial cells by non-typable *Haemophilus influenzae* and *Streptococcus pneumoniae* which are major bacterial pathogens in acute exacerbations of COPD. In addition, we found that a PAFr antagonist decreased the adhesion of both respiratory bacterial pathogens to non-cigarette exposure control levels. This highlights the possibility that epithelial receptors, that are upregulated in response to cigarette smoke, could be targeted to specifically block chronic bacterial infections of the lower respiratory tract. In this commentary, we explore the question of whether adhesion to a temporally-upregulated host receptor is a common event in chronic bacterial disease, and as such, could represent a putative therapeutic target for blocking infection by respiratory and other pathogens.

## Chronic obstructive pulmonary disease and platelet-activating factor receptor

Lung disease remains a major cause of death globally after heart disease and stroke [1]. Bacterial infection of the respiratory tract drives a number of communicable diseases including pneumonia and tuberculosis, but also acute exacerbations (AE) of the non-communicable illness, chronic obstructive pulmonary disease (COPD). The first step in bacterial colonisation of the respiratory tract involves the binding of a bacterial adhesin to its cognate receptor presented on the surface of the host cell. Expression of the microbial receptors on host epithelium is therefore, key to the establishment of a respiratory infection.

Non-typable *Haemophilus influenzae* (NTHi) and *Streptococcus pneumoniae* are the main bacterial species involved in triggering AECOPD [2, 3]. The primary risk factor for the development of COPD is cigarette smoke [4, 5]. Expression of a G-protein coupled receptor (GPCR) protein, Platelet-activating factor receptor (PAFr), is significantly up-regulated in the airway epithelium of both the large and small airways in smokers and COPD patients [6, 7].

## Adhesion of NTHi and *S. pneumoniae* to upregulated bronchial epithelial PAFr

In a new publication, we report that attachment of both NTHi and *S. pneumoniae* to bronchial epithelial cells is enhanced by exposure to cigarette smoke extract [8]. We identified that the increased bacterial adherence correlated with upregulation of expression of PAFr on the surface of the lung epithelial cells [8]. PAFr is known to bind to phosphorylcholine which is a component of the cell wall of the above bacterial species and is a molecular mimic of human platelet-activating factor [9]. While host receptors are often regarded as being available perpetually for bacterial adhesion, it is apparent from our work that NTHi and *S. pneumoniae* utilise a receptor which is temporally-upregulated in response to a specific external stimulus, in this case, tobacco smoke. We also determined that a PAFr antagonist, WEB-2086 (Apafant), reduced the adhesion of both respiratory bacterial pathogens down to non-cigarette exposure control levels [8]. This highlights the possibility that host surface receptors, that are upregulated in response to cigarette smoke, could be targeted to specifically block chronic infections of the lower respiratory tract by both NTHi and *S. pneumoniae*.

## Role of PAFr and the effects of PAFr antagonism

PAFr, when bound to its natural ligand, platelet activating factor (PAF), has been implicated in the pathophysiology of anaphylaxis, bronchial hyper-responsiveness, endotoxin shock, respiratory bacterial infections, skin diseases, and disorders of the central nervous system [10]. In addition to blocking PAFr-mediated signaling, a number of PAFr-antagonists have been reported to inhibit enzymes such as acetylcholinesterase [11], and both cyclooxygenase and lipoxygenase [12], thus, disrupting the inflammatory cascade. Long-term administration of a PAFr-antagonist (L659989) significantly delayed the onset of proteinuria and improved survival in a mouse model of glomerulonephritis [13]. Clinical trials in humans, primarily focused on asthma, have been performed with PAFr antagonists. SR27417 inhibited PAF-induced symptoms (coughing and dyspnoea) in asthma patients with only minor side effects [14]. Similarly, PAF analogue CV-3988, was not associated with any major adverse events in humans at doses of 750–2000 μg/kg [15]. In terms of returning to pre-treatment levels, no clinically evident adverse effects were reported for PAFr antagonist BN52021 nearly one year after a clinical trial in asthmatic children [16].

## Alternate examples of temporally-induced receptors for bacterial adhesion

Use of a non-constitutively expressed surface receptor for colonisation appears to be opportunistic. The question therefore arises: Is this phenomenon unique to PAFr and respiratory pathogens NTHi and *S. pneumoniae*?

Some strains of *Pseudomonas aeruginosa*, a major bacterial pathogen in cystic fibrosis, also express ChoP [17]. Infection of A549 human airway epithelial cells and mouse lungs with ExoU-expressing *P. aeruginosa* results in upregulation of PAFr expression which is inhibited in the presence of PAFr antagonist WEB-2086 [18]. Instances of upregulation of other receptors of microbial adhesion on host cells can also be found. For example, NTHi stimulates expression of intercellular adhesion molecule 1 (ICAM-1) on respiratory epithelial cells in an inoculum-dependent manner [19, 20]. ICAM-1 in turn is a receptor for NTHi adhesion and therefore, NTHi appears to upregulate the expression of its own receptor. In relation to the digestive system, a similar mechanism is observed with respect to induction of expression by Crohn’s Disease-associated *Escherichia coli* of its own ileal epithelial cell receptor, carcinoembryonic antigen-related cell adhesion molecule 6 (CEACAM6) [21].

It is also evident that infection by one pathogen can increase the level of a receptor available for another pathogen. For example, it was reported recently that influenza A viral (IAV) infection of human lung epithelial cells results in heightened expression of cellular adhesins which in turn promotes streptococcal infection [22]. A previously unrecognised mechanism was identified in which IAV neuraminidase was found to promote the expression of fibronectin and α5 integrin through the activation of transforming growth factor beta (TGF-β) leading to increased adhesion to respiratory epithelial cells by *Streptococcus pyogenes* [22]. Thus, influenza infection effectively “primes” the respiratory epithelium for subsequent colonisation by Group A *Streptococci* by enhancing receptor presentation.

In a similar manner, respiratory syncytial virus (RSV) infection of respiratory epithelial cells has been shown to elevate the expression of ICAM-1, PAFr and CEACAM1, which correlated with increased adhesion of NTHi and *S. pneumoniae* [23]. NTHi binding to the lung epithelial cells was inhibited by the addition of either anti-ICAM-1 or anti-PAFr antibodies, while adhesion of *S. pneumoniae* was blocked in the presence of anti-PAFr antibodies [23]. An additional surface receptor which may be presented during RSV infection consists of the RSV-encoded G glycoprotein which, when artificially expressed in respiratory epithelial cells, increased adherence by NTHi and *S. pneumoniae* [24]. Rhinovirus infection has also been shown to significantly increase fibronectin, PAFr and CEACAM expression in nasal epithelial cells correlating with augmented adhesion of *S. aureus*, *S. pneumoniae*, and *H. influenzae* [25]. The upregulation of PAFr expression due to rhinovirus infection of respiratory epithelial cells is mediated by the nuclear transcription factor-κB (NFκB) [26]. It has been suggested that the elevated PAFr expression in airway epithelial cells due to viral infection may contribute to the development of pneumonia after rhinovirus infection [26]. Furthermore, with approximately 25 % of acute exacerbations of COPD (AECOPD) exhibiting viral/bacterial co-infection [27], viral induction of bacterial receptor presentation on respiratory epithelium could potentially play a role in bacterial colonisation during AECOPD.

## Conclusions and future perspectives

Our recent findings [8] should be examined in the context of their translation into new ways to manage lung illnesses caused by bacterial infection. Given its up-regulation in the respiratory epithelium in response to cigarette smoke and viral infection, PAFr represents a putative anti-infective target for therapeutics against AECOPD, and pneumonia, caused by NTHi or *S. pneumoniae*. PAFr antagonists including WEB-2086 are well tolerated [28–30] and therefore, provide chemical scaffolds for the synthesis of new classes of antibacterial drugs. The upregulation of bacterial epithelial receptors is not unique to cigarette exposure but is also seen following infection with a number of viral respiratory pathogens including influenza, RSV and rhinovirus. It is therefore apparent that bacterial pathogens can “abduct” the upregulation of specific epithelial cell receptors for their adhesion, with the effect that it boosts their infectivity at a preferred host site, including the respiratory tract. Other receptor targets such as CAECAM and ICAM-1 should also be examined with regard to the generation of new anti-infective agents for the treatment of AECOPD and pneumonia.

In conclusion, blocking adhesion to temporally upregulated host cell receptors may offer a basis for developing drugs that prevent infection by respiratory and other pathogens following exposure to insults such as viral infection or cigarette smoking.

## Abbreviations

AE: acute exacerbations
COPD: chronic obstructive pulmonary disease
GPCR: G-protein coupled receptor protein
NTHi: non-typable *Haemophilus influenzae*
PAFr: platelet-activating factor receptor
ICAM-1: intercellular adhesion molecule 1
CEACAM: carcinoembryonic antigen-related cell adhesion molecule
